# Supplementary material for: How Asian Breast Cancer Patients Experience Unequal Incidence of Chemotherapy Side Effects: A Look at Ethnic Disparities in Febrile Neutropenia Rates
Source: Cancers (Basel). 2023 Jul 12;15(14):3590. doi: 10.3390/cancers15143590 (PMC10377556; doi:10.3390/cancers15143590)
Supplement: Supplementary file 1 [file cancers-15-03590-s001.zip › Supplementary Figures S1 and S2.pdf]

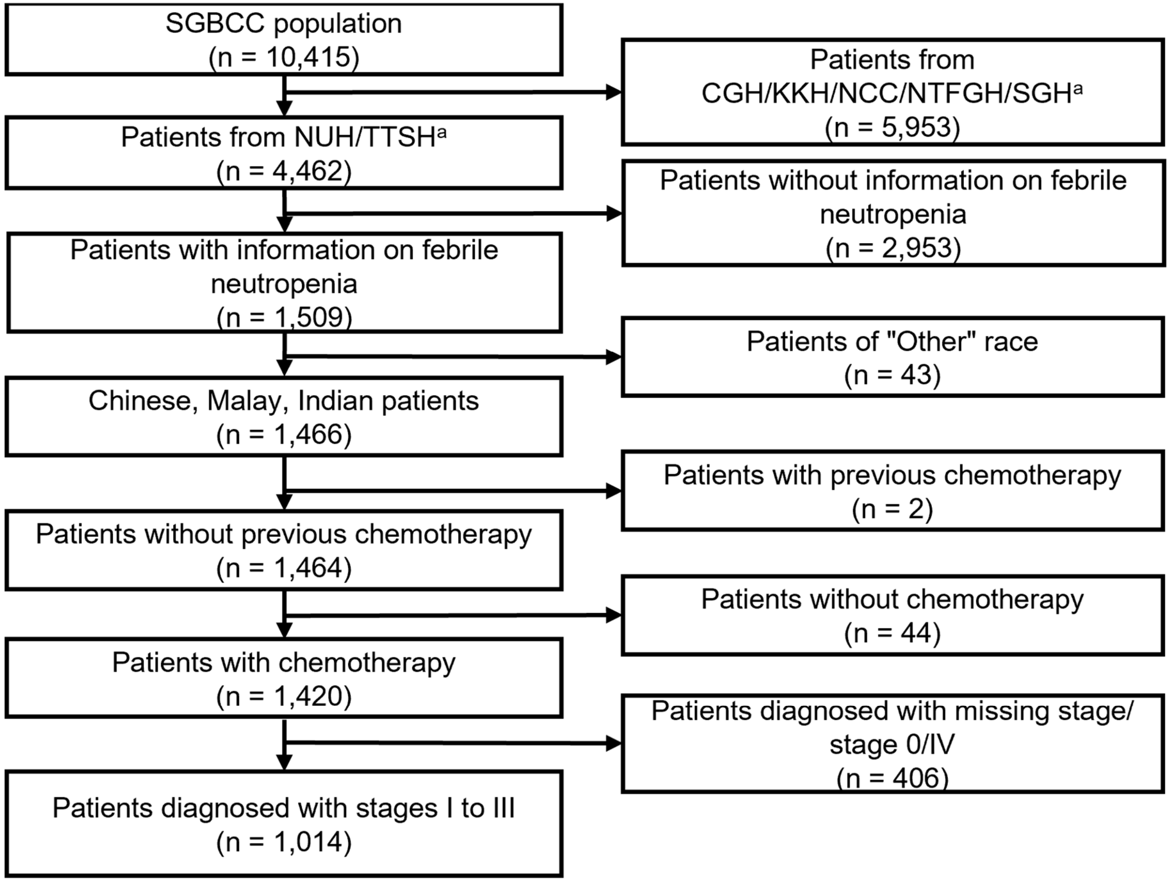

[a]NUH=National University Hospital, KKH=KK Women's and Children's Hospital, TTSH= Tan Tock Seng Hospital, NCCS=National Cancer Centre Singapore, SGH=Singapore General Hospital, CGH=Changi General Hospital, NTFGH=Ng Teng Fong General Hospital.

Supplementary Figure S1. Flow chart of SGBCC population.

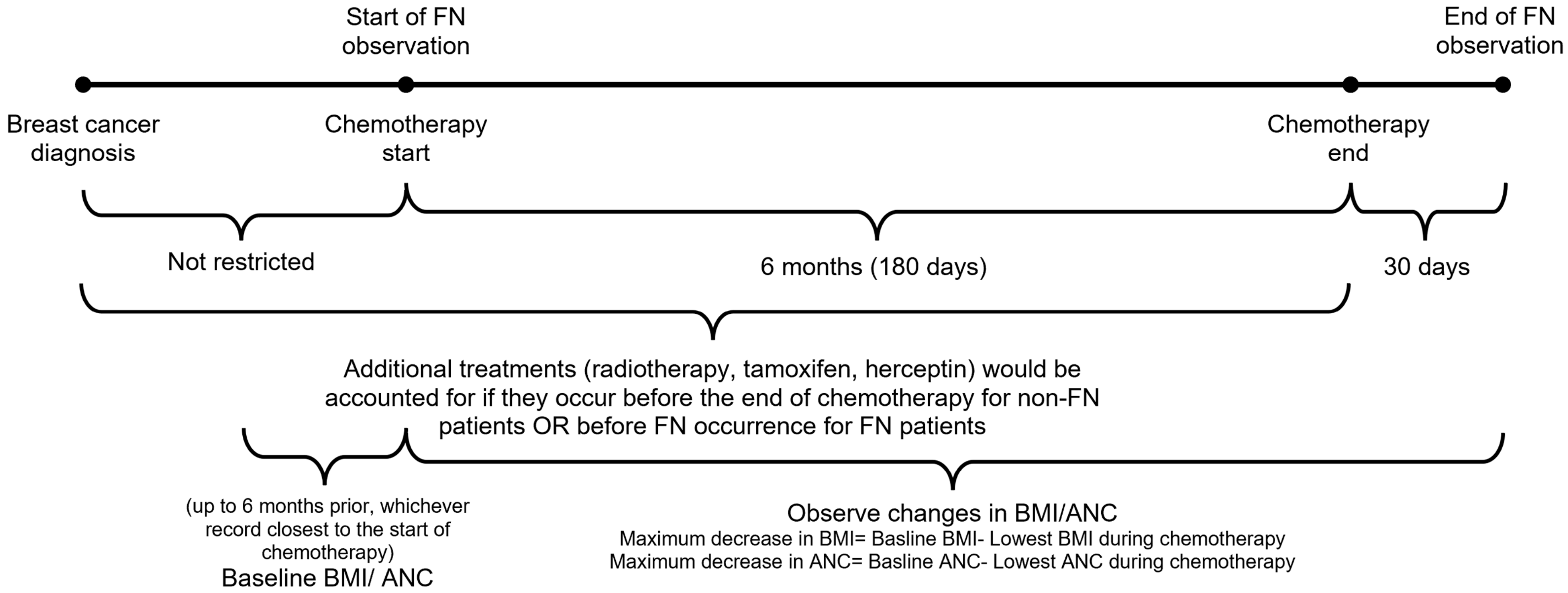

Supplementary Figure S2. Schematic of study time points.
